# Issues pertaining to PET imaging of liver cancer

**Published:** 2014-08-06

**Authors:** Zhenghong Lee, Guangbin Luo

**Affiliations:** 1 Professor of Radiology, Case Western Reserve University, Cleveland, OH, USA; 2 Associate professor of Genetics, Case Western Reserve University, Cleveland, OH, USA

## Abstract

Positron emission tomography (PET) imaging using 2-deoxy-2-[F-18]fluoro-D-glucose (FDG) has proven valuable in the diagnosis, staging and restaging for many cancers. However, its application for liver cancer has remained limited owing in part to the relatively high background uptake of the tracer in the liver plus the significant variability of the tumor specific uptake in liver cancer among patients. Thus, for primarily liver cancer, in particular, hepatocellular carcinoma (HCC), radio-tracers with better tumor-enhancing uptake/retention are still sought in order to harness the great power of PET imaging. Here, we reviewed some recent investigations with lipid-based small molecule PET radio-tracers with relevance to fasting, and discuss their potential in the diagnosis and staging of HCCs.

[F-18]-FDG-based PET imaging (FDG-PET) has proven valuable in the diagnosis, staging and restaging for many cancers. To date, this medical imaging modality has represented an important and powerful tool in oncology practice. In particular, it has been approved by the Centers for Medicare and Medicaid Services for the diagnosis, initial staging, and restaging of non–small cell lung cancer, colorectal cancer, Hodgkin and non-Hodgkin lymphoma, esophageal cancer, melanoma, head and neck cancers, and breast cancer as well as characterization of solitary pulmonary nodules. The fundamental basis for the remarkable success of this technology lies at the simple fact that the uptake of glucose is greatly enhanced in many types of cancers because of the up-regulated glycolytic pathway of glucose metabolism in these cancers. Food intake impacts the glycolytic pathway and thus FDG-PET scans. In humans, glucose uptake is highly regulated in the vast majority of tissue/organ systems. Importantly, under a fasting condition, the levels of glucose uptake in the cells from non-brain tissue/organ systems are quite low. However, upon oncogenic transformation (up-regulated glycolysis), the levels of glucose uptake for many of these transformed cells are elevated. Since FDG is a glucose analog, its uptake in the cells of many tumors are also elevated. Moreover, FDG has a unique hydroxyl group at the 2’ position of the glucose that prohibits its utilization by hexokinase. Thus, once taken up by these cells, FDG is converted to FDG-6-phosphate, a metabolite that cannot be metabolized further and hence are accumulated within the tumor cells. This enhanced tumor-cell specific accumulation of radiolabeled FDG metabolite under a fasting state therefore offers a readily image-able tumor specific signal, enabling the detection of the tumor as well as the quantitative evaluation of its progression status via PET imaging.

Several types of tumors, including primary cancer of the liver, such as HCC, exhibit great heterogeneities in terms of tumor specific PET imaging signal with FDG-PET, i.e. variations in tumor-specific FDG accumulation (1). The underlying basis for the variations in FDG accumulation in these tumor cells has remained unknown to date. In the case of HCC, it likely reflects, at least partly, the diversified metabolic features of individual patients. Fasting is essential for any meaningful PET imaging studies with FDG. Otherwise, the PET image quality would be considerably degraded (2). Fasting is intended to lower patients’ blood sugar level so that FDG would not compete with glucose for its transporters for tumor uptake during the PET scan (3). Fasting also reduces insulin levels and thus lowers the activity of glucose transporters in the surrounding tissues and muscles to further enhance FDG uptake in the tumors relative to its surroundings (4). However, some patients have conditions in which fasting alone does not bring the blood glucose level down, and a mandatory check of the glucose level is enforced before FDG-PET scans in some clinics. Therefore, in order to hardness the power of the concept of using PET for detecting and staging HCCs, the development of tracers other than FDG appear to be an unmet need.

Non-FDG PET tracers such as [C-11]-acetate and [C-11]-choline or F-18 labeled fluorinated choline tracers have shown uptake in HCC (7, 8). The entrapped radio-labeled metabolites of the original tracers responsible for their uptake are mainly phospholipids as these tracers are incorporated along the lipid synthesis pathways (9, 10). Is pre-imaging fasting required for these lipid-based small molecule tracers? Currently, there is no clear guideline from either European Association of Nuclear Medicine or Society of Nuclear Medicine and Molecular Imaging regarding this. In our pre-clinical studies, intra-subject comparison was made for acetate and choline tracers with and without fasting to evaluate the effect of fasting on tracer uptake in HCC for PET imaging. Preliminary results from these studies showed that fasting has little impact on the uptake of acetate and choline tracers in HCC (11, 12). Why is this, in light that lipogenesis and glycolysis are connected.

For normal hepatocytes in a well-fed state, excessive glucose and its metabolites are converted into acetyl-CoA for fatty acid synthesis into fat storage. In a fast state, fatty acids are mobilized, and metabolized into acetyl-CoA, and then converted into ketone bodies and transported outside liver to be the energy sources for vital organs. How are the liver cancer cells different from the surrounding hepatocytes during fed or fast state? The main difference between hepatocytes and HCC cells seems to be the “enhanced lipogenesis”. Most cancers demonstrate up-regulation in aerobic glycolysis whether it is in the fed or fast state. There is often a shift from oxidation to synthesis halfway through this up-regulated glycolytic pathway in cancer as shown in Figure 1. That is the so-called glucose-dependent de novo lipogenesis (13), in which excessive pyruvate from glycolysis would enter mitochondria (truncated TCA cycle) to be converted into acetyl-CoA and then into citrate; citrate transporter will carrier citrate out of mitochondria along the concentration gradient into cytosol where ATP Citrate Lyase (ACL) will convert citrate into cytosolic acetyl-CoA (14), which is incorporated into lipogensis. The “enhanced” portion comes from the up-regulated alternative or salvage lipogenic pathways that tumor cells use to produce acetyl-CoA from acetate by way of acetyl-CoA synthetase (ACAS), independently of ACL, which happened especially in early stages of HCC. This lack of regulatory control for lipogensis including fatty acid and/or cholesterol synthesis in liver cancer has been noticed (15). The impaired feedback mechanism made liver cancer different from its surround tissues for lipid-tracer metabolism. Consequently, the acetyl-CoA de novo lipogenesis has no apparent impact on the integration of radio-acetate into phospholipids during time span of a PET scan. The requirement of pre-imaging fast can thus be relaxed.

For choline-based tracers, the pathway to phospholipids is through either CDP-choline pathway in HCC or PE methylation pathway in hepatocytes (10), and does not seem to be influenced by blood glucose level or glycolysis. In our pre-clinical studies, however, the fed state included a diet consistent with standard animal care; whether a high fat diet will have a transient impact on PET imaging with lipid-based tracers is yet to be investigated. Nevertheless, the initial finding from these studies is of significance for clinical use of radiolabeled acetate or choline tracers for HCC imaging. Further validation studies in humans with HCC using these lipid-based tracers as well as the effect of fasting can provide additional assurance on all important aspects of routine clinical practice.

## Figures and Tables

**Figure 1:**
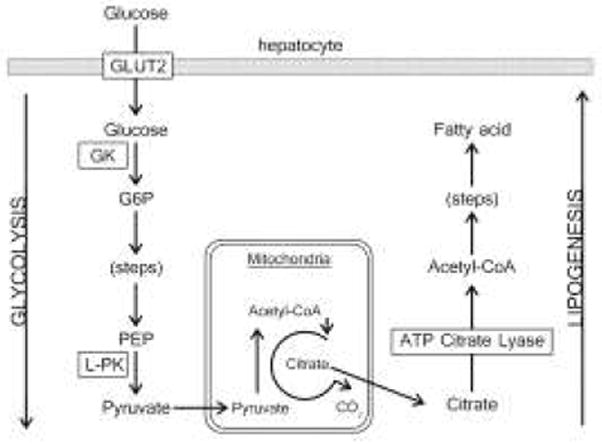
Glucose-dependent lipogenesis connecting the two pathways.

## References

[1] Kostakoglu L, Agress H, Goldsmith SJ (2003). Clinical role of FDG PET in evaluation of cancer patients. Radiographics.

[2] Lindholm P, Minn H, Leskinen-Kallio Bergman SJ, Ruotsalainen U, Joensuu H (1993). Influence of the blood glucose concentration on FDG uptake in cancer - a PET study. J Nucl Med.

[3] Lee KH, Ko BH, Paik JY (2005). Effects of anesthetic agents and fasting duration on 18F-FDG biodistribution and insulin levels in tumor-bearing mice. J Nucl Med.

[4] Okumura W, Iwasaki T, Toyama T (2004). Usefulness of fasting 18F-FDG PET in identification of cardiac sarcoidosis. J Nucl Med.

[5] Jeng LB, Changlai SP, Shen YY, Lin CC, Tsai CH, Kao CH (2003). Limited value of 18F-2-deoxyglucose positron emission tomography to detect hepatocellular carcinoma in hepatitis B virus carriers. Hepatogastroenterology.

[6] Khan MA, Combs CS, Brunt EM, Lowe VJ, Wolverson MK, Solomon H, Collins BT, Di Bisceglie AM (2000). Positron emission tomography scanning in the evaluation of hepatocellular carcinoma. J Hepatol.

[7] Ho CL, Yu SC, Yeung DW (2003). 11C-acetate PET imaging in hepatocellular carcinoma and other liver masses. J Nucl Med.

[8] Talbot JN, Gutman F, Fartoux L (2006). PET/CT in patients with hepatocellular carcinoma using [(18)F]fluorocholine: preliminary comparison with [ (18)F]FDG PET/CT. Eur J Nucl Med Mol Imaging.

[9] Salem N, Kuang Y, Corn DJ, Erokwu B, Kolthammer JA, Tian H, Wu C, Wang F, Wang Y, Lee Z (2011). [(Methyl) 1-11C]-Acetate Metabolism in Hepatocellular Carcinoma. Molecular Imaging and Biology.

[10] Kuang Y, Salem N, Tian H, Kolthammer JA, Corn DJ, Wu C, Wang F, Wang Y, Lee Z (2011). Imaging Lipid Synthesis in Hepatocellular Carcinoma with [Methyl-11C]Choline Correlated with Metabolites Study in vivo. Journal of Nuclear Medicine.

[11] Kolthammer JA, Corn DJ, Tenley N (2011). PET imaging of hepatocellular carcinoma with 18F-fluoroethylcholine and 11C-choline. Eur J Nucl Med Mol Imaging.

[12] Tenley N, Corn DJ, Yuan L, Wu C, Lee Z (2013). The effect of fasting on PET Imaging of Hepatocellular Carcinoma. Journal of Cancer Therapy.

[13] Hatzivassiliou G, Zhao F, Bauer DE, Andreadis C, Shaw AN, Dhanak D, Hingorani SR, Tuveson DA, Thompson CB (2005). ATP citrate lyase inhibition can suppress tumor cell growth. Cancer Cell.

[14] Alberts B, Johnson A, Lewis J (2002). Cell chemistry and biosynthesis. Molecular Biology of the Cell.

[15] Elwood JC, Morris HP (1968). Lack of adaptation in lipogenesis by hepatoma 9121. J Lipid Res.

